# Physiological and Behavioral Evaluation of Shelter Dogs During Veterinary Routine Health Checks

**DOI:** 10.3390/vetsci12060583

**Published:** 2025-06-13

**Authors:** Valentina Gazzano, Maria Claudia Curadi, Paolo Baragli, Chiara Mariti, Francesca Cecchi, Stefano Cavallo, Luigi Sacchettino, Angelo Gazzano

**Affiliations:** 1 Department of Veterinary Sciences, University of Pisa, 56124 Pisa, Italy; valentina.gazzano@unipi.it (V.G.); maria.claudia.curadi@unipi.it (M.C.C.); paolo.baragli@unipi.it (P.B.); chiara.mariti@unipi.it (C.M.); francesca.cecchi@unipi.it (F.C.); ste.cavallo@gmail.com (S.C.); angelo.gazzano@unipi.it (A.G.); 2 Department of Veterinary Medicine and Animal Production, University of Naples Federico II, 80137 Naples, Italy

**Keywords:** hearth rate, stress, oxytocin, cortisol, serotonin, interleukin-6

## Abstract

Shelter environments can be inherently stressful for dogs, a highly social species that forms strong attachment bond with humans. This study evaluated stress responses in 26 shelter dogs during routine veterinary examinations, analyzing behavioral scores alongside physiological and hormonal parameters, including heart rate, body temperature, cortisol (CRT), oxytocin (OXT), serotonin (5-HT), tryptophan (TRP), and interleukin-6 (IL-6). A significant negative correlation was observed between OXT and CRT (ρ = –0.540, *p* = 0.007), particularly in dogs exhibiting relaxed behavior. OXT was also negatively correlated with body temperature (ρ = –0.435, *p* = 0.034), supporting its potential role in modulating stress-induced hyperthermia. No significant associations were found between TRP, 5-HT, IL-6, or other physiological measures and behavioral scores. The absence of correlation between TRP and 5-HT may be due to blood–brain barrier regulation, while IL-6′s lack of association suggests further investigation is needed to clarify its role in canine stress responses. These findings highlight OXT’s possible buffering effect on the hypothalamic–pituitary–adrenal axis and suggest that behavioral assessment may offer a more sensitive measure of canine stress than hormonal or physiological parameters alone. Future studies with larger and more diverse samples are needed to confirm and expand upon these results.

## Simple Summary

Life in a shelter can be highly stressful for dogs, who are inherently social animals and form strong attachment bonds with humans. In this study, we investigated the responses of 26 shelter dogs, who had been housed in a facility for periods ranging from 1 month to 9 years (median duration: 12 months), to a routine veterinary examination. We wanted to understand whether their behavior during the visit matched up with signs of stress, such as changes in heart rate, body temperature, or hormone levels. We found that one hormone in particular, oxytocin, often called the “bonding hormone,” was linked to lower levels of cortisol, the main stress hormone. Oxytocin also seemed to help keep body temperature down, which often rises when animals are stressed. Other substances we measured, like serotonin, tryptophan and interleukin-6, did not show clear links to behavior in this setting. These results suggest that oxytocin might help dogs manage stress during challenging moments, especially when they feel safe or are treated gently. Overall, observing a dog’s behavior appears to be one of the most reliable ways to assess how stressed they are, possibly more than measuring hormones alone.

## 1. Introduction

The process of domestication has transformed the dog into a companion animal capable of forming strong attachment bonds with humans [1,2]. This deep interspecies connection has contributed to a widespread perception of dogs as “pets,” prompting many countries to prohibit their euthanasia except under specific, regulated circumstances. In Italy, Law No. 281/91 bans the euthanasia of stray and ownerless dogs and mandates the creation of public or private shelters to house these animals while they await adoption (www.normattiva.it (accessed on 1 February 2025)).

However, the implementation of this “no-kill” policy has resulted in a considerable number of dogs remaining unadopted and living out their lives in shelters [3]. Prolonged confinement in such environments is often associated with behavioral deterioration, including increased restlessness and stereotypic behaviors [4]. Behavioral problems are, also, among the leading causes of dog relinquishment and abandonment [5,6,7] and represent a major barrier to successful adoption [8]. Additional limiting factors include advanced age [9] and aesthetic preferences for specific breeds or coat colors [8]. Moreover, aging and associated health conditions, such as neurodegenerative diseases like canine cognitive dysfunction, further reduce adoption potential [9,10,11].

The psychophysical welfare of dogs in shelters is shaped by a complex interplay of intrinsic factors (e.g., age, behavioral or metabolic disorders) and extrinsic factors (e.g., shelter management, housing environment, and prior experiences) [4]. Comprehensive evaluation and, where necessary, improvement of these factors are crucial not only for maintaining animal welfare but also for increasing the likelihood of adoption.

In this context, regular veterinary examinations, coupled with behavioral assessments conducted by professionals specialized in animal behavior, serve as essential tools for the early detection of both pre-existing and shelter-induced psychophysical conditions. These evaluations also facilitate the implementation of individualized behavioral rehabilitation programs aimed at improving adoption outcomes.

Nevertheless, routine handling, even when performed by trained professionals, may itself be a source of stress for animals unfamiliar with close human contact, potentially undermining their overall well-being [12,13,14,15,16]. Previous research has demonstrated that shelter dogs exhibit less approach and physical contact behaviors toward strangers than pet dogs [17].

Understanding the biological and behavioral responses to such stress is therefore essential for interpreting animal welfare status and adjusting management practices accordingly.

Stress is a multifactorial response that occurs when an organism perceives a threat to its homeostasis [18]. In animals, acute stress can be assessed using a combination of behavioral observations, physiological parameters (such as heart rate and body temperature), and biological markers measured in blood and saliva [19]. As in other organisms, systemic stressors, actual or perceived threats to survival, trigger a coordinated physiological response aimed at restoring homeostasis [20]. The autonomic nervous system responds first, followed by the activation of the hypothalamic–pituitary–adrenal (HPA) axis, which initiates a cascade of hormonal and neurochemical events, often accompanied by measurable behavioral changes [21].

Among the physiological responses associated with HPA axis activation, increases in heart rate and body temperature are commonly used as measurable indicators of acute stress. The latter, known as stress-induced hyperthermia, is partly mediated by the release of interleukin-6 (IL-6), a cytokine that plays a key role in the crosstalk between the immune and neuroendocrine systems [22]. IL-6 is secreted by lymphocytes and macrophages in response to stress-induced HPA axis activation. Experimental studies in rodents have shown that acute stressors, such as the open field test, result in elevated circulating IL-6 levels alongside increased body temperature [22]. Similarly, in clinical settings, Shuttler et al. [23] have reported significantly elevated IL-6 levels in dogs hospitalized for severe inflammatory conditions, such as systemic inflammatory response syndrome or pancreatitis, with concentrations exceeding 400 pg/mL often associated with poor prognosis or mortality.

Cortisol (CRT) remains the primary hormone involved in the physiological stress response and can be quantified in blood and saliva samples in acute events or in hair samples for chronic events [24,25]. Its hematic and salivary concentrations typically increase rapidly following acute stress events; however, CRT levels may also rise due to physical exertion, potentially reflecting general arousal rather than psychological distress [26].

Another hormone involved in the regulation of the stress response is oxytocin (OXT), a neuropeptide produced in specific hypothalamic nuclei. OXT modulates the activity of the HPA axis and plays a central role in social behavior and emotional regulation. Research in both humans and non-human animals has demonstrated that OXT can reduce HPA axis activation, dampen inflammatory responses, and alleviate anxiety-related behaviors [27]. In humans, an inverse relationship between OXT and CRT levels has been documented [28]. In addition to its role in maternal behavior, OXT facilitates positive social interactions and prosocial behaviors [29]. In contexts involving calm and affiliative interactions, both intraspecific and interspecific, OXT levels tend to rise while CRT levels decrease [28,30]. However, this correlation is less well established in dogs, as some studies have reported increased CRT levels even during positive interactions with humans [31].

The activation of the HPA axis also influences behavior, which can be systematically assessed using ethogram-based scoring systems. For instance, Mills et al. [32] investigated the efficacy of a pheromone diffuser in reducing stress in dogs during veterinary visits by employing a structured ethogram. A refined behavioral scoring system was applied in which each dog was assigned a score based on observable stress-related behaviors: 1 = relaxed, 2 = mild stress signals (e.g., lip-licking, head-turning, crouched posture, trembling), 3 = tension without growling, 4 = tension with growling, 5 = escape attempts, and 6 = aggression attempts.

Furthermore, several studies have highlighted associations between behavioral changes and fluctuations in neurotransmitter levels, particularly serotonin (5-HT). This monoamine plays a crucial role in the regulation of mood, cognition, and behavior across mammalian species [33]. Both central and peripheral serotonin concentrations are closely linked to the availability of tryptophan (TRP), an essential amino acid required for 5-HT synthesis in the brain and gut [34,35,36]. Low blood 5-HT levels have been correlated with increased aggression [37,38], whereas higher levels have been associated with anxiety-like states [39].

Against this background, the present study aims to assess the psychophysical condition of shelter dogs in response to routine veterinary handling.

We hypothesize that there could be a correlation between behavioral responses and selected physiological and hormonal indicators of stress. Additionally, this study represents the first attempt to investigate the role of interleukin-6 (IL-6) as a potential biomarker of stress in dogs.

## 2. Materials and Methods

### 2.1. Subjects

Twenty-six mixed-breed dogs (8 females and 18 males; age range: 6 months to 15 years; mean age: 6.1 ± 3.51 years; no one neutered) were included in the study. All dogs had been living in a shelter for a minimum of 1 month and up to a maximum of 9 years (median duration: 12 months). The sample was drawn from two public shelter facilities: 10 dogs were housed at the Pisa shelter (“Soffio di Vento,” Via di Granuccio, 56121 Pisa, Italy), and 16 dogs at the Lucca shelter (“Pontetetto,” Via Santeschi, 993, 55100 Lucca, Italy). Both facilities were managed by the same non-profit organization, the “Ponteverde” social cooperative (Piazza Vittime dei Lager Nazisti, 3, 56025 Pontedera, PI, Italy), and followed comparable routines and management protocols. Each year, the Department of Veterinary Sciences, in accordance with an agreement established with the shelter management, conducts health check-ups on a selected number of dogs. This clinical examination, including also a complete blood count (CBC), is aimed at evaluating the dogs for parasitic diseases and overall health status.

The dogs were housed in either single or double kennels (four dogs kept in pairs at the shelter in Lucca and two at the shelter in Pisa). Each kennel provided access to both indoor and outdoor areas. The front side of each kennel was made of wire mesh, allowing the dogs to have visual contact with those housed directly opposite. In front of the kennels was a corridor where each dog was allowed individual free access (except for those already housed in pairs) for one hour daily. Additionally, volunteers took the dogs outside the facility for a walk twice a week, with each walk lasting approximately one hour. Feeding occurred twice daily, at 8:00 a.m. and 4:00 p.m., using the same commercial dry food across both shelters.

Prior to the study, none of the dogs had undergone a comprehensive behavioral assessment. Subjects exhibiting defensive or offensive aggression toward humans, posing a potential safety risk to the personnel or requiring sedation for handling, were excluded. Similarly, dogs presenting physical conditions that could affect behavioral responses to handling, including neurological, orthopedic, dermatological disorders, or recent injuries, were not included. All dogs had received at least one veterinary check-up, either at the time of shelter admission or, if in good health, prior to their annual vaccination.

### 2.2. Experimental Setting and Procedure

The study was conducted over a five-week period between October and December 2024. On the day of the procedure, a shelter volunteer retrieved the selected dog from its kennel and escorted it to the examination room within the facility. There, a veterinarian performed a general medical assessment to confirm the dog was in good health. The same veterinarian conducted all examinations across both facilities to ensure consistency.

Each examination lasted approximately 20 min per dog and followed a standardized sequence: fitting the dog with a muzzle, placing it on the examination table, assessing the conjunctival mucosa, palpating the lymph nodes, otoscopic examination of the ear canals, measuring rectal temperature, and auscultating, with a stethoscope, the lung and the heart, while simultaneously detecting heart rate. Following the examination, a venous blood sample was collected. Dog behavioral responses to entering the room, restraint, handling, and blood collection were observed by a certified veterinary behaviorist, consistent across all subjects. The observation period began with the dog’s entry into the room designated for veterinary visits, before placing the muzzle and then ending with the dog’s removal from the room after blood sampling. Dogs were scored using a behavioral scale adapted from Mills et al. [32], as described in the introduction. The overall score reflected the dog’s behavior throughout all phases of the veterinary procedure. In cases where a dog displayed aggressive behavior, a score of 6 was assigned for all subsequent stages of the examination. When a dog displayed behaviors spanning multiple categories, the highest applicable score was assigned by the behaviorist to capture the most intense response observed.

Both the detailed scoring system and a simplified classification into two groups—Group 1 (score = 1, relaxed) and Group 2 (score > 1, indicative of stress-related behaviors)—were used for statistical analysis.

### 2.3. Blood Collection, Storage and Analysis

Blood samples were collected from the cephalic vein approximately 3–4 h after the dogs’ morning meal. One aliquot of whole blood was treated with EDTA and used for CBC analysis. A second aliquot, without EDTA, was used to assess serum concentrations of 5-HT, total TRP, CRT, OXT and IL-6.

Samples were refrigerated during transportation to the laboratory. For CBC testing, samples were kept at room temperature for 30–60 min prior to analysis with Mindray BC-2800vet auto hematology analyzer (Mindray Building, Keji 12th Road South, High-tech Industrial Park, Nanshan, Shenzhen 518057, P.R. China). For serum analyses, samples were centrifuged using an ALC 4237R refrigerated centrifuge (ALC International S.r.l., Milan, Italy) at 7000 rpm, with the temperature gradually reduced from 20 °C to 4 °C. The resulting serum was divided into 200 µL aliquots and stored at −20 °C until analysis. To assess concentrations of 5-HT, total TRP, CRT, OXT and IL-6 serum samples were left to thaw at room temperature for 30 min.

The extraction and quantification of 5-HT and TRP in serum were performed using high-performance liquid chromatography (HPLC) with fluorometric detection, following the procedure described by Riggio and colleagues [34]. Data acquisition was carried out using Jasco Borwin v.1.5.0 software (Jasco Corporation, Ishikawa-machi Hachioji-shi, Tokyo, Japan), with JMBS HERCULE 2000 VI.0 serving as the electronic interface between the chromatographic instruments and the data acquisition system.

Serotonin creatinine sulfate monohydrate and L-tryptophan standards were purchased from Sigma-Aldrich Inc. (Saint Louis, MO, USA). Stock solutions of 5-HT (10 mM) and TRP (100 mM) were prepared in 10 mL of 10% perchloric acid (HClO₄), aliquoted into 1 mL portions, and stored at −20 °C. Daily working standard solutions were prepared by diluting the stock solutions in 4% HClO₄ and were used to identify chromatographic peaks and generate calibration curves.

Serum concentrations of OXT, CRT, and IL-6 were measured using the Cayman Chemical Oxytocin ELISA Kit (Item #500440, Ann Arbor, MI, USA), Cayman’s Cortisol ELISA Kit (Item #500360, Ann Arbor, MI, USA), and the Canine IL-6 ELISA Kit (RAB0525, Millipore, Saint Louis, MO, USA), respectively. Optical density values were read within 15 min using a standard plate reader (Multiskan™, Thermo Fisher Scientific, Waltham, MA, USA) at a wavelength of 450 nm.

### 2.4. Statistical Analysis

Statistical analyses were performed using GraphPad Prism 9 (Graph-Pad Software, San Diego, CA, USA). Data normality was assessed, and because not all data were normally distributed, Spearman’s correlation was performed. The presence of correlations was assessed among the following variables: age, heart rate, body temperature, behavioral score, 5-HT, CRT, OXT, and IL-6.

## 3. Results

Table 1 shows the values of the different physiological and hormonal markers analyzed, as well as the behavioral score attributed to the different subjects. Regarding the behavioral evaluation, 11 dogs received a score of 1, 4 dogs a score of 2, 6 dogs a score of 3, 2 dogs a score of 4, and 2 dogs a score of 5, while only one dog achieved the highest score of 6. Body temperature values ranged from 37.4 °C to 39.6 °C. The minimum and maximum values for the biochemical variables analyzed were as follows: 5-HT (serotonin), 0.21–1.46 µM/L; TRP (tryptophan), 8.7–29.1 µM/L; CRT (cortisol), 0.85–79.87 ng/mL; OXT (oxytocin), 14.01–303.64 ng/mL; and IL-6 (interleukin-6), 0.009–3.06 ng/mL.

All dogs included in the study had normal CBC.

**Figure 1:**
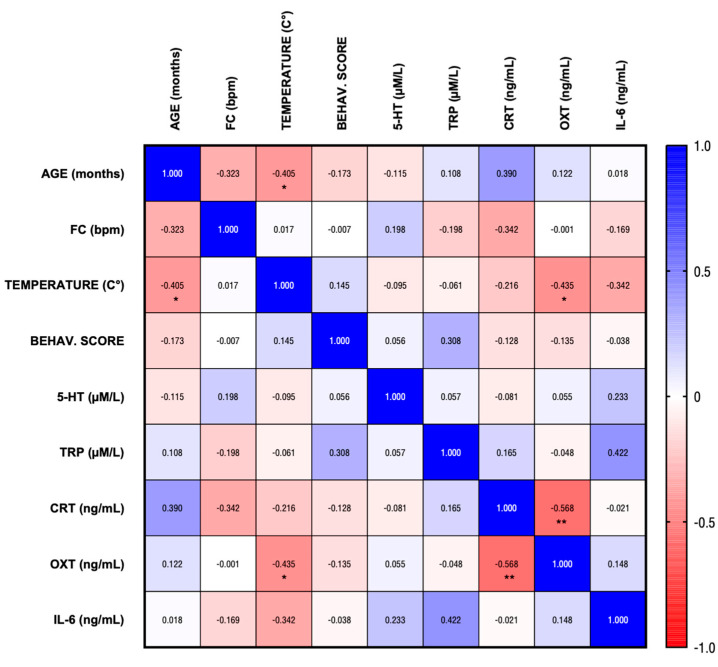
Matrix of Spearman’s correlation: ρ = +1 indicates a perfect positive correlation, ρ = −1 indicates a perfect negative correlation, and ρ = 0 indicates no correlation. (* *p* ≤ 0.05, ** *p* ≤ 0.01).

Figure 1 shows a heatmap of Spearman’s correlation coefficients (ρ) among the analyzed variables, based on the classification of the dogs into six behavioral score categories. The color gradient represents the strength and direction of the correlations, with blue indicating positive correlations and red indicating negative correlations. Significant correlations are indicated with an asterisk (*, *p* ≤ 0.05) or two asterisks (**, *p* ≤ 0.01). A strong significant negative correlation was observed between OXT and CRT (ρ = −0.540, *p* = 0.007). Additionally, another significant negative correlation was found between temperature and OXT (ρ = −0.435, *p* = 0.034), as well as between temperature and age (ρ = −0.41, *p* = 0.040). No significant correlations were found among the other parameters and no significant differences were identified in the parameters based on sex.

By dividing the dogs into two groups based on their behavioral score (group 1 = score 1 and group 2 = score higher than 1), the negative correlation between blood levels of OXT and CRT is maintained for group 1 (Spearman’s ρ = −0.733; *p* = 0.020) but not for group 2 (Spearman’s ρ = −0.44; *p* = 0.155). No other statistically significant correlations were found for the remaining parameters within the two groups of animals. The comparison of biomarker levels between the two groups did not reveal any statistically significant differences.

## 4. Discussion

Life in a kennel can be inherently stressful for a highly social species like the dog, who forms strong attachment bonds with humans from an early age [40]. In shelters, interaction with humans becomes limited, although the presence of volunteers in many facilities ensures a minimal level of social contact. However, this reduction in regular human interaction may lead to a gradual desocialization of the animal, diminishing its ability to cope with potentially stressful experiences such as routine veterinary handling [41].

Our study aimed to investigate potential stress responses in shelter dogs undergoing routine veterinary examinations. To achieve this, we measured a range of physiological and hormonal parameters and analyzed their correlation with behavioral assessments. During veterinary check-ups, these animals may display a diminished ability to utilize coping strategies, which can lead to elevated stress levels.

Behavioral observation is one of the most commonly used approaches to assess animal welfare. As stress can significantly alter canine behavior [42], it is considered a reliable indicator of welfare status [43]. A detailed behavioral assessment can therefore help identify whether an animal is experiencing a negative emotional state, such as those triggered by interactions with conspecifics or humans. In such encounters, dogs communicate emotional tension through visual signals and specific behaviors, which can be interpreted by canine behavior specialists to gauge the intensity of the stress experienced.

The study of visual communication in domestic dogs has its roots in the observation of their ancestor, the wolf. However, dogs show marked differences in social behavior, particularly concerning aggression, when compared with wolves [44,45]. According to Scott [46], most dog breeds have a higher threshold for aggressive responses than wolves. Fox [47] has noted that wolves, during intraspecific interactions, may exhibit specific behaviors aimed at interrupting the interaction, even when it is aggressive. These were referred to as “cut-off signals” [48].

Turid Rugaas [49] identified several behaviors in domestic dogs that appear to serve a similar function, interrupting or de-escalating potentially aggressive encounters. Rugaas suggested that these so-called “calming signals” might be even more effective than cut-off signals in wolves, as they help to prevent conflict before aggression escalates [49]. Similar behaviors, often described in the literature using terms such as “appeasement behaviors” [50,51,52], are always emitted even during interactions of dogs with humans [53]. A scientific evaluation of the effectiveness of these behaviors was conducted by Mariti and colleagues [54], who observed that behaviors such as freezing, head-turning, looking away, and reducing body size were among the most frequently exhibited, either individually or in combination, during dog-to-dog encounters. These findings suggest that such signals are indicative of stress and may play a crucial role in de-escalating aggression, even after an aggressive display has already been initiated.

The scale developed by Mills and colleagues [32] considers a range of stress-related indicators, including calming signals such as lip-licking, head-turning, crouched posture, and trembling—classified as signs of mild stress (score 2)—as well as freezing behavior without growling (score 3). When applied by a trained and experienced observer, as in the present study, this scale provides an objective framework for interpreting canine behavior during clinical examinations.

Previous studies have attempted to correlate behavioral indicators with physiological and hormonal markers of stress in dogs, yet results have often been inconclusive [34]. Our findings revealed a significant negative correlation between OXT and CRT levels. Interestingly, this relationship was only maintained in dogs belonging to Group 1—those exhibiting calm and positive behavior. This observation aligns with existing literature suggesting that OXT fosters social interactions, and its levels increase following positive human-animal contact [28,30,55].

However, research in other species has produced mixed outcomes. While OXT appears to inhibit the HPA axis and reduce anxiety-related behaviors in laboratory animals and humans [56], a study in dogs has reported a positive correlation between OXT and CRT levels in non-aversive human-dog interaction [31]. Previous research has demonstrated that a brief, positive interaction lasting three minutes between a dog and its owner can lead to a rapid increase in blood OXT levels, peaking within the first three minutes and returning to baseline within approximately 30 min [31]. In our study, interactions between the dog and the handler occurred continuously during the 20 min leading up to blood collection. It is therefore plausible that OXT secretion was more prolonged in our subjects, even though the measured hormone levels were lower than those reported in previous studies [31]. Notably, the timing of our blood sampling coincided with the reported peak in blood CRT, which has been shown to occur around 15 min after a three-minute positive human–dog interaction [31].

Our data suggest that elevated OXT levels, particularly in dogs experiencing a positive emotional state, may help mitigate stress by limiting CRT increases. Though handling and restraint during a veterinary visit are inherently stressful, the negative relationship between OXT and CRT supports the hypothesis that OXT may buffer the stress response. This hypothesis is supported by a recent study by Barrera and colleagues [17], in which intranasal oxytocin (OXT) was administered to both pet and shelter dogs. While OXT had no significant effect on sociability tests, it did increase the duration of gaze directed at the human face during the extinction phase of the test. As this phase is known to elicit stress-like emotional responses [57], the anxiolytic properties of oxytocin may help sustain this social behavior. Further studies are needed to determine whether intranasal OXT could serve as an effective tool in supporting the behavioral rehabilitation of shelter dogs.

Moreover, this idea is further sustained by the observed negative correlation between OXT and body temperature. In fact, stress is known to cause “stress-induced hyperthermia,” an increase in body temperature driven by sympathetic nervous system activation [58]. Further, the average heart rate recorded (101.2 ± 20.94 bpm ± S.D.) remained within the physiological range for dogs, suggesting a generally effective coping response to the stressor.

Another notable negative correlation emerged between body temperature and age, consistent with prior studies in both humans and other mammals indicating that thermoregulation becomes less efficient with age due to reduced metabolic activity [59,60,61].

No significant correlations were found between serum levels of TRP, 5-HT, or other physiological and hormonal markers. Although a positive association between TRP and 5-HT in the brain is well established [62], this relationship was not observed in peripheral blood, likely due to the restrictive action of the blood–brain barrier. This barrier not only regulates TRP transport into the brain but also limits the passage of 5-HT from the central nervous system to the periphery. While low peripheral TRP levels have been linked to aggressive behavior in several mammalian species, including dogs [63], such associations often emerge in studies involving dietary manipulation. In our study, the absence of dietary intervention may have contributed to the limited TRP variability and the lack of behavioral effects, reinforcing the idea that TRP’s influence on behavior depends largely on its availability to the brain and its impact on central 5-HT synthesis [64].

Consistent with previous findings [65] our results show no significant variation in serum 5-HT levels among dogs with different behavioral profiles. While previous studies have linked low 5-HT to fear or aggression [38,39,66], those cases typically involved animals with diagnosed behavioral disorders. This suggests that 5-HT may only play a meaningful role in behavior when underlying emotional or psychological vulnerabilities are present [67]. In contrast, only one dog in our study (score 6) displayed aggressive behavior when restraint during blood sampling became more intense. This can be attributed to the fact that dogs with evident aggressive tendencies were excluded from the study a priori.

IL-6 levels in the bloodstream are known to rise in response to acute psychological stress, as shown in laboratory animals subjected to stressors like open field exposure [22], foot shocks, restraint, and conditioned aversive stimuli [68]. IL-6 promotes corticotropin release hormone (CRH) release from the hypothalamus in a dose-dependent manner [69,70], and, even in the absence of CRH, it can still stimulate ACTH release, likely through direct action on corticotroph receptors. In humans, subcutaneous IL-6 administration also leads to dose-dependent increases in ACTH and CRT [71]. Additionally, OXT has been shown to inhibit IL-6 secretion in vitro, significantly reducing its release from activated macrophages and endothelial cells [72].

Our research represents the first attempt to explore IL-6′s role in stress mechanisms in dogs, and we did not identify any significant correlations with the physiological and hormonal markers measured. Additional studies will be necessary to determine whether OXT exerts an inhibitory effect on IL-6 release in vivo, as observed in vitro.

This study has several limitations, which, however, do not diminish the relevance of the findings.

Although the living conditions of the dogs in the two shelters are very similar, a small number of animals (six dogs) are housed in pairs rather than individually. Due to the limited sample size, it was not possible to determine whether this housing condition influenced the variables under study. Further research is needed to assess whether housing dogs in pairs or small groups in shelters can enhance their welfare.

The variability in hormonal responses among the dogs, along with the inability to measure certain variables in some subjects, suggests that a larger sample size would be necessary to draw more robust and generalizable conclusions.

Moreover, the sample population lacked sufficient diversity to cover all behavioral categories, particularly severe fear or aggression; while this may have affected our findings, it also likely reflects the behavioral characteristics of the general dog population.

Finally, the uneven sex distribution (8 females and 18 males) may explain the absence of any observable sex-related differences.

## 5. Conclusions

The assessment of canine welfare, particularly for dogs housed in shelters, must include an evaluation of their behavior, especially in relation to humans. Stimulating positive emotional states in dogs appears to be a promising strategy for enhancing their coping abilities in response to stressful events. OXT, due to its role in promoting prosocial behavior, emerges as a key hormone in facilitating adaptive coping mechanisms and in mitigating CRT elevation during stress.

Other hormonal parameters did not show significant correlations with behavioral scores. TRP and 5-HTwere not significantly correlated, either with each other or with other stress indicators, likely reflecting the complexity of their metabolic pathways. Although IL-6 was examined in relation to both stress biomarkers and behavioral responses for the first time in this context, no significant associations were found. Further research is needed to better understand the intricate interplay between these physiological markers and their roles in the canine stress response.

## Figures and Tables

**Table 1:** The table reports the levels of the different physiological and hormonal markers analyzed: heart rate (beats per minute), rectal body temperature (°C), serum CRT (ng/mL), OXT (ng/mL), interleukin-6 (ng/mL), serotonin (5-HT, µM/L), and total tryptophan (TRP, µM/L); n.d. = not detected.

Dog ID	Sex	Age (Months)	Heart Rate (BPM)	Temp. (C°)	Behav. Score	5-HT (µM/L)	TRP (µM/L)	CRT (ng/mL)	OXT (ng/mL)	IL-6 (ng/mL)
1	F	72	78	39.0	1	0.48	29.1	47.41	51.99	0.010
2	M	120	90	38.2	1	1.09	16.8	49.3	99.43	0.45
3	M	180	96	38.5	1	0.73	19.1	16.29	92.4	0.42
4	M	48	118	39.6	1	0.36	11.5	38.83	42.06	0.14
5	M	36	90	38.3	1	0.82	11.1	48.1	34.83	0.38
6	F	72	150	37.9	1	n.d.	n.d.	n.d.	61.35	n.d.
7	M	24	144	39.1	1	1.28	12.4	8.53	55.9	0.09
8	F	120	84	38.6	1	0.91	21.7	49.81	22.69	0.78
9	M	108	138	38.6	1	1.03	8.7	71.36	21.82	0.011
10	F	24	132	38.6	1	0.47	10.1	0.85	225.77	0.21
11	F	96	72	38.5	1	0.77	14.3	10.12	303.64	0.67
12	M	6	90	39.5	2	1.46	13.5	n.d.	14.01	0.061
13	M	96	84	39.0	2	0.63	18.6	41.08	39.2	0.42
14	M	96	78	38.7	2	0.33	9.1	37.28	81.66	0.012
15	M	60	101	38.0	2	0.96	24.5	74.56	82.97	n.d.
16	M	84	100	39.0	3	1.28	12.4	0.87	107.96	0.06
17	F	36	98	39.5	3	0.21	20.0	31.94	14.04	n.d.
18	M	84	102	39.1	3	0.63	18.6	71.23	14.03	n.d.
19	F	24	118	38.3	3	1.04	23.9	0.88	90.83	3.06
20	M	132	104	37.4	3	0.52	15.7	24.67	71.72	0.009
21	M	132	78	38.2	3	0.69	17.7	79.87	40.63	0.22
22	F	36	84	38.5	4	n.d.	n.d.	n.d.	71.09	n.d.
23	M	36	96	39.2	4	0.52	15.7	n.d.	n.d.	n.d.
24	M	60	102	38.2	5	0.89	15.0	35.5	45.01	0.45
25	M	60	96	39.1	5	0.91	21.7	43.39	n.d.	n.d.
26	M	60	108	39.4	6	1.17	20.1	0.89	42.56	n.d.

## Data Availability

The data presented in this study are available on request from the corresponding author.
